# Complete Genome Sequences of the Endophytic *Streptomyces* sp. Strains LUP30 and LUP47B, Isolated from Lucerne Plants

**DOI:** 10.1128/genomeA.00556-17

**Published:** 2017-06-15

**Authors:** Christopher M. M. Franco, Eric M. Adetutu, Hoang X. Le, Ross A. Ballard, Ricardo Araujo, Shanan S. Tobe, Bobby Paul, Sandeep Mallya, Kapaetu Satyamoorthy

**Affiliations:** a Medical Biotechnology, School of Medicine, Flinders University, Adelaide, Australia; b South Australian Research and Development Institute, Adelaide, Australia; c Instituto de Investigação e Inovação em Saúde (i3S) and IPATIMUP, Institute of Molecular Pathology, Universidade do Porto, Porto, Portugal; d School of Biological Sciences, Flinders University, Adelaide, Australia; e Department of Chemistry and Physics, Arcadia University, Glenside, Pennsylvania, USA; f School of Life Sciences, Manipal University, Manipal, India

## Abstract

The complete genome sequences of two endophytic *Streptomyces* sp. strains, LUP30 and LUP47B, were analyzed. These strains were isolated from surface-sterilized roots of lucerne plants from South Australia and were found to promote the growth of the rhizobial partner *in vitro* and significantly increased nodulation and nitrogen fixation in lucerne plants.

## GENOME ANNOUNCEMENT

Lucerne (*Medicago sativa* L.) is an important perennial legume extensively cultivated for hay, silage, and pasture in Australia and around the world. It is estimated that 35 million ha of lucerne are cultivated annually in about 80 countries (1). Lucerne is nodulated by and forms effective symbiotic nitrogen-fixing associations with rhizobia (*Sinorhizobium* spp.), thereby providing significant fixed-nitrogen inputs into agricultural systems (2). Endophytic actinobacteria have also been isolated from lucerne roots and have been shown to promote root nodulation and contribute to increased symbiotic nitrogen fixation and improved legume growth (3, 4). The phylum *Actinobacteria* is important in plant (roots) interactions with other ecosystem components, with *Streptomyces* being a widely recognized genus in this phylum. *Streptomyces* spp. are ubiquitous and have been isolated from soil (rhizosphere and nonrhizosphere) and within different parts of plants, such as leaves, stems, and roots (5). Their presence in legume roots as endophytes has been shown to promote nodulation and improve plant growth and N content (4, 6, 7). In addition, members of this genus have been used as biocontrol agents and to enhance drought resistance in crops (8, 9).

In this study, the complete genome sequencing of *Streptomyces* sp. strains LUP30 and LUP47B was carried out on an Illumina MiSeq sequencing platform (AGRF, Sydney, Australia), generating 724 Mb of data. *De novo* assembly of reads was performed using A5-miseq (10) and resulted in 131 and 56 scaffolds for LUP30 and LUP47B, respectively. However, the largest scaffold in LUP30, which was 452,038 bp in size, was smaller than the one in LUP47B (1,520,852 bp). In addition, LUP30 had 436 subsystems, 651 noncoding gene RNAs, a G+C content of 71.6%, and 7,600 coding sequences in a genome of 9,184,130 bp. LUP47B, in contrast, had 452 subsystems, 633 noncoding genes, 70.3% G+C content, and 9,421 coding sequences in a genome of 10,998,901 bp. The Rapid Annotations using Subsystems Technology (RAST) server was used for gene prediction and annotation, and the SEED-viewer platform was used for the curation of genomic data (11). A comparison of the *Streptomyces* genomes with the RAST genomes database revealed that the closest neighbor for LUP30 was *Streptomyces coelicolor* (genome ID 100226.1) and for LUP47B, it was *Streptomyces avermitilis* (genome ID 227882.11). Further analysis of the 16S rRNA gene revealed the nearest-neighbor type strains for strain LUP30 to be that of *Streptomyces rishiriensis*, whereas for LUP47B, it is those of *Streptomyces ciscaucasicus* and *Streptomyces canus*.

### Accession number(s).

The complete genome sequences of *Streptomyces* LUP30 and LUP47B have been deposited in DDBJ/ENA/GenBank under the accession numbers MJAJ00000000 and MJAH00000000, respectively, and the corresponding versions described in this paper are MJAJ01000000 and MJAH01000000.
